# Associations of maternal PM2.5 exposure with preterm birth and miscarriage in women undergoing *in vitro* fertilization: a retrospective cohort study

**DOI:** 10.3389/fendo.2025.1460976

**Published:** 2025-01-27

**Authors:** Miaoxin Chen, Qiaoyu Chen, Gengze Liao, Chunyan Sun, Cong Liu, Xia Meng, Wentao Li, Andong Qiu, Orhan Bukulmez, Haidong Kan, Feng Wang, Lap Ah Tse, Xiaoming Teng

**Affiliations:** ^1^ Centre for Assisted Reproduction, Shanghai Key Laboratory of Maternal Fetal Medicine, Shanghai Institute of Maternal-Fetal Medicine and Gynecologic Oncology, Shanghai First Maternity and Infant Hospital, School of Medicine, Tongji University, Shanghai, China; ^2^ JC School of Public Health and Primary Care, the Chinese University of Hong Kong, Hong Kong, Hong Kong SAR, China; ^3^ School of Public Health, Key Lab of Public Health Safety of the Ministry of Education, NHC Key Lab of Health Technology Assessment, IRDR ICoE on Risk Interconnectivity and Governance on Weather/Climate Extremes Impact and Public Health, Fudan University, Shanghai, China; ^4^ Department of Obstetrics and Gynecology, Monash Medical Centre, Monash University, Melbourne, VIC, Australia; ^5^ Division of Reproductive Endocrinology and Infertility, Department of Obstetrics and Gynecology, The University of Texas Southwestern Medical Center, Dallas, TX, United States

**Keywords:** PM2.5 exposure, preterm birth, miscarriage, particulate matter, *in vitro* fertilization

## Abstract

**Background:**

Excessive exposure to PM2.5 can be detrimental to reproductive health. The objective of this study was to investigate the potential associations between ambient PM2.5 exposure during different periods and negative pregnancy outcomes, such as miscarriage and preterm birth, in patients who underwent assisted reproductive technology (ART).

**Methods:**

This retrospective cohort study examined the outcomes of 2,839 infertile women aged ≤ 45 years who underwent their first fresh or frozen-thawed embryo transfer at the Shanghai First Maternity and Infant Hospital between April 2016 and December 2019. Satellite data were used to determine the daily average levels of PM2.5, and exposure was categorized as excessive if it exceeded the WHO’s interim target 2 level of 50 µg/m^3^. The analysis was conducted separately for seven different periods. Our study used multinomial logistic regression models to explore the potential associations between PM2.5 exposure and adverse pregnancy outcomes. Sensitivity analysis was conducted by excluding women who underwent blastocyst transfer.

**Results:**

Daily PM2.5 exposure exceeding the threshold (50 µg/m^3^) was associated with an increased risk of miscarriage during the period after confirmation of clinical pregnancy or biochemical pregnancy, with adjusted odds ratios (AORs) of 2.22 (95% CI 1.75-2.81) and 2.23 (95% CI 1.68-2.96), respectively. Moreover, for each increase of 10 µg/m^3^ above the threshold for PM2.5, there was a 46% elevated risk of preterm birth (AOR = 1.46, 95% CI 1.09-1.94) during the period after the confirmation of clinical pregnancy and a 61% elevated risk of preterm birth (AOR = 1.61, 95% CI 1.16-2.23) during the period after the confirmation of biochemical pregnancy. Our stratified analyses revealed that women with an endometrial thickness <11 mm or who underwent frozen embryo transfer were more vulnerable to PM2.5 exposure, leading to higher rates of preterm birth.

**Conclusion:**

Excessive PM2.5 exposure after biochemical pregnancy or clinical pregnancy was associated with increased risks of preterm birth and miscarriage among women who underwent ART.

## Graphical Abstract

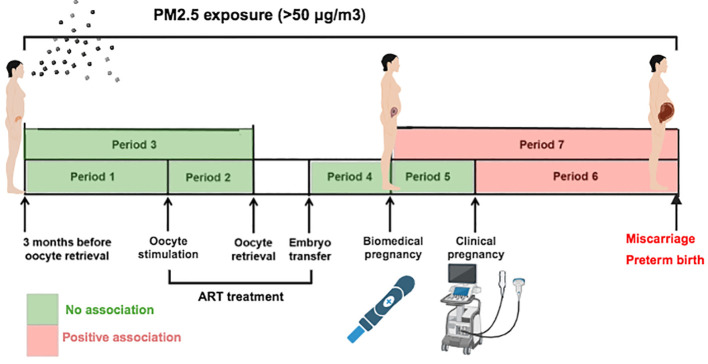

## Introduction

Ambient fine particulate matter with an aerodynamic diameter ≤ 2.5 μm (PM2.5) poses a health threat to populations worldwide and has detrimental effects on multiple organs and systems, including the reproductive system (1, 2). In addition, physiological changes during pregnancy make pregnant women particularly susceptible to the negative health impacts of PM2.5 (3). Previous research has indicated a link between PM2.5 and potential risks to fecundability and fertility (4, 5), as well as unfavorable pregnancy outcomes such as miscarriage (6), fetal growth restriction (7), low birth weight, and preterm birth (3, 8, 9). However, cohort studies regarding PM2.5 exposure and pregnancy outcomes were mainly conducted in women who conceived naturally (2, 7, 8). However, there is a gap in knowledge concerning the potential link between PM2.5 exposure and adverse pregnancy outcomes among women undergoing ART treatment.

The estimated rate of infertility among couples of reproductive age in China is as high as 25%, which exceeds the global average of 15% (10). An increasing number of infertile couples are undergoing ART treatments to conceive and have children, with in vitro fertilization (IVF) being the most commonly utilized method (11). Previous studies have demonstrated that women who conceive through IVF may be more vulnerable to ambient air pollution, including PM2.5 exposure, than women who conceive naturally (12), with a decreased probability of oocyte yield, clinical pregnancy and live birth among women with high ambient air pollution during pregnancy (11, 13–21). Limited research has been conducted on the potential link between PM2.5 exposure and adverse pregnancy outcomes, including preterm birth and pregnancy loss, in women undergoing ART.

Preterm birth and pregnancy loss, including miscarriage and biochemical pregnancy loss, are major adverse pregnancy outcomes of IVF treatment for patients who have a positive human chorionic gonadotropin (hCG) test (22). Nevertheless, there is a lack of research on the relationship between exposure to PM2.5 and preterm birth and/or pregnancy loss in women undergoing IVF, and the findings are inconclusive (12, 19, 23). In a prospective study of women in the United States, chronic daily PM2.5 exposure (mean concentration of 8.8 μg/m^3^) from the date of the first positive hCG test until the date of pregnancy loss or live birth was not significantly associated with pregnancy loss (19). The lack of a significant association might be due to the lower concentration of PM2.5 exposure, which was within the normal level of the WHO’s 24-hour air quality guidelines (PM2.5 < 15 μg/m^3^) (19, 24). In contrast, another prospective cohort study in China showed a significant increase in the likelihood of biochemical pregnancy loss for every 10 μg/m^3^ increase in PM2.5 exposure from the time of hCG testing to 30 days after embryo transfer. The average daily PM2.5 exposure of patients in this study was 45.4 μg/m^3^ (25). The available evidence on the link between preterm birth and excessive PM2.5 exposure is scarce. A retrospective study in Hangzhou of China, revealed that daily PM2.5 exposure at a mean level of 36.0 μg/m^3^ was significantly associated with an increased risk of preterm birth in all periods of pregnancy (from 85 days before oocyte retrieval to delivery outcome) among the ART population (23). However, a national cohort study in China reported that an increased risk of preterm birth in newborns conceived by ART was only linked to excessive PM2.5 exposure (median 55.0 μg/m^3^) during the third trimester (12).

Therefore, the current understanding of the association between PM2.5 exposure and adverse pregnancy outcomes, such as preterm birth and pregnancy loss, in women undergoing IVF is incomplete and uncertain. Moreover, there is insufficient evidence to determine which stage of pregnancy after IVF treatment is most susceptible to adverse pregnancy outcomes resulting from high levels of PM2.5 exposure. Our study aimed to investigate the effects of PM2.5 exposure on adverse pregnancy outcomes (i.e., pregnancy loss and preterm birth) between different pregnancy periods among women who underwent ART.

## Methods

### Study design and subject recruitment

**Figure 1:**
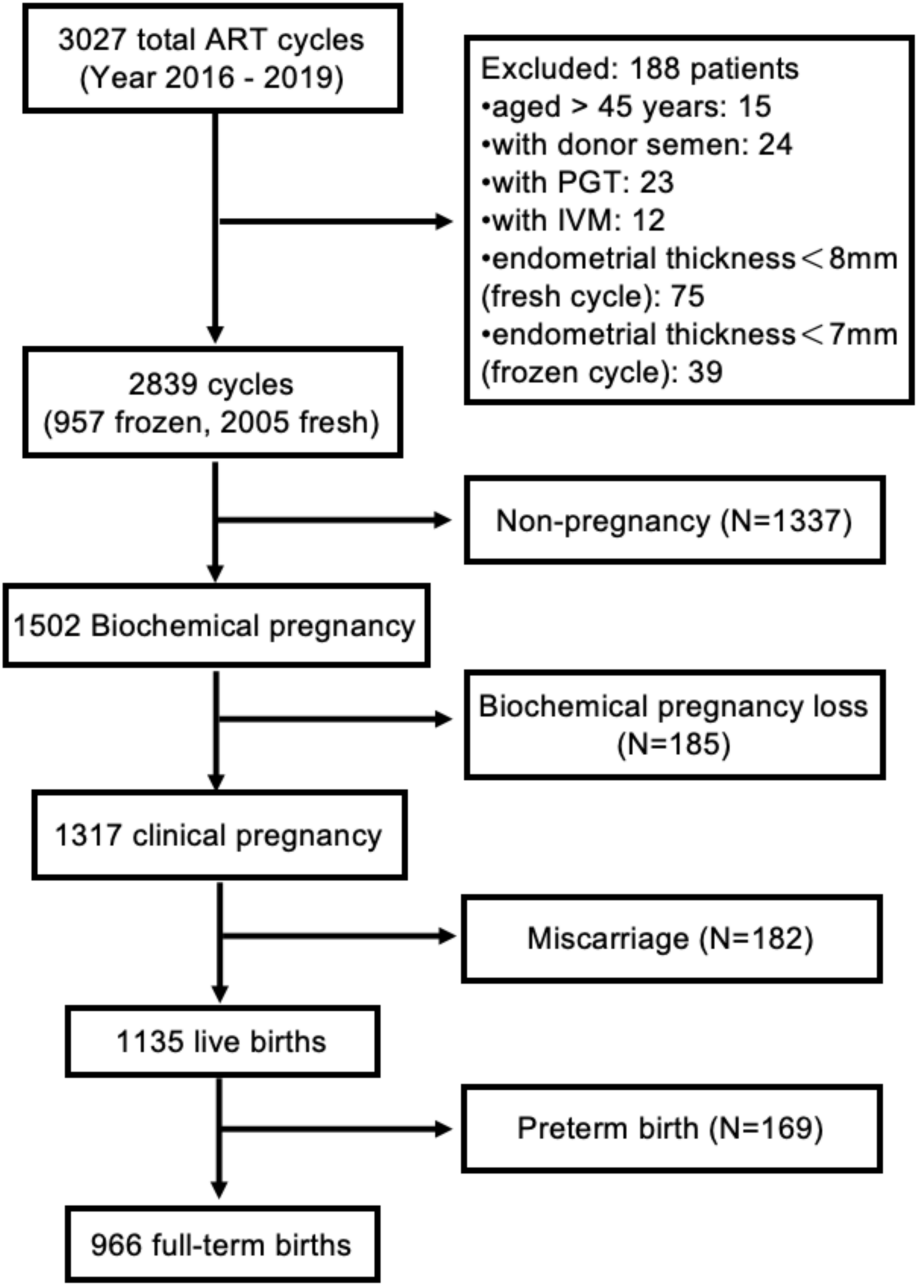
Subject recruitment and follow-up.

This was a retrospective cohort study. The subjects of this study were women residing in Shanghai who were transferred their first fresh or frozen embryos at the Centre for Assisted Reproduction of Shanghai First Maternity and Infant Hospital from April 2016 to December 2019. The following patients who underwent ART at our center were not included in this study: (1) were aged more than 45 years, (2) used donor semen, (3) underwent preimplantation genetic testing, (4) exhibited oocyte maturation *in vitro*, (5) had an endometrial thinness <8 mm with fresh embryo transfer, and (6) had an endometrial thinness <7 mm with frozen embryo transfer. **Figure 1** shows the flowchart outlining the recruitment and follow-up of the study participants. This study received approval from the Research Ethics Committee of Shanghai First Maternity and Infant Hospital (KS22298).

### ART procedures

The center followed a standard operating procedure (SOP) to administer controlled ovarian stimulation (COS) treatment to all participants. The details of the COS protocol were described previously (21). ART procedures, including semen preparation, conventional IVF or intracytoplasmic sperm injection (ICSI) processes, embryo culture, and assessment, were performed as described previously (26). According to the Center’s SOP, women who underwent fresh embryo transfer received one or two high-quality embryos three or five days after oocyte retrieval with the guidance of transabdominal ultrasound. Luteal phase support was initiated on the day of oocyte retrieval (27). The procedures for endometrial preparation and luteal phase support in frozen-thawed embryo transfer have been previously described (21). Women with a positive hCG test continued to receive luteal-phase support until ten weeks of pregnancy.

### Outcome definition and covariate data collection

The presence of biochemical pregnancy was determined by a serum hCG level exceeding 10 mIU/ml two weeks after embryo transfer (16). The term biochemical pregnancy loss refers to an early pregnancy loss that does not develop into a clinical pregnancy (28). All pregnant women were followed up until delivery or miscarriage. Clinical pregnancy was indicated upon the identification of a gestational sac through ultrasound four weeks after embryo transfer. Miscarriage was defined as the spontaneous loss of an intrauterine pregnancy before the 20th week of gestation (29). Live birth was defined as the delivery of one or more living infants with a gestational age of 20 weeks or more or a birth weight exceeding 1,000 g (30). Delivery between 20 and 37 weeks of gestation was classified as preterm birth, while delivery at 37 weeks of gestation or later was considered full-term birth (31).

As previously described, we retrieved covariate data from medical records at the Centre, which included information on the age of the females, age of the males, body mass index (BMI), address, level of education, employment status, duration and causes of infertility, COS method, duration of ovarian stimulation, dosage of gonadotropin stimulation used, progesterone level on the trigger day, total number of oocytes retrieved, method used for fertilization, thickness of the endometrial lining on the day the embryo was transferred, year and season of embryo transfer, number of transferred embryos and whether the transferred embryos were fresh or frozen (21).

### Definition of periods of IVF and pregnancy

**Figure 2:**
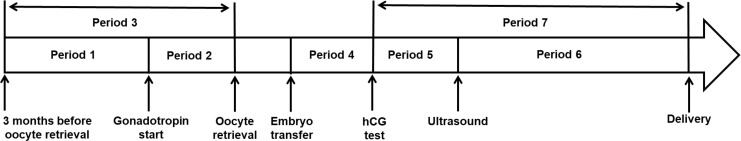
Period definition of IVF stages and pregnancy.

As shown in **Figure 2**, we used 7 periods of IVF treatment and pregnancy to study the effects of daily exposure to PM2.5 on the outcomes of IVF therapy. The periods were as follows: period 1 included the time between the 3 months prior to oocyte retrieval and gonadotropin-induced ovarian stimulation; period 2 included the time between gonadotropin-induced ovarian stimulation and oocyte retrieval; period 3 included the total 3 months preceding oocyte retrieval; period 4 included the time from embryo transfer to the time of serum hCG testing; period 5 followed from the hCG serum test to the time of ultrasound testing to confirm intrauterine pregnancy; period 6 included the time of ultrasound testing to the time of delivery or miscarriage; and finally, period 7 included the whole period between the hCG serum testing stage and the time of delivery or miscarriage.

### Assessment of ambient PM2.5 exposure

Following an established method (31, 32), we used a satellite-based model with a spatial resolution of 1x1 km to estimate the daily concentrations of PM2.5 during each of the seven periods studied. This was done utilizing a random forest algorithm where the PM2.5 measurements of >1,500 ground-based monitoring sites between 2013 and 2019 were used as the dependent variable, and the multiangle implementation of atmospheric correction (MAIAC) aerosol optical depth (AOD) retrieval data were used as the independent variables. We also utilized additional predictors, including population density, land use data, meteorological variables, and the Modern-Era Retrospective Analysis for Research and Applications (MERRA-2) PM2.5 products. In terms of missing AOD data, we approached this issue through a gap-filling method where estimated PM2.5 data were generated by combining findings from models with and without AOD data. Each participant had their daily mean PM2.5 concentration assigned by connecting the 1x1 km grid data to their residential address geocodes (32).

### Statistical analysis

One-way ANOVA was utilized to assess variations among continuous variables that followed a normal distribution, while the Kruskal−Wallis test was used for continuous variables with a skewed distribution. The chi-square test and Fisher’s exact test were applied to assess the categorical variables between different IVF/ICSI outcomes or time periods.

The average daily PM2.5 concentration among our participants across the study period was 36.9 µg/m^3^, which is close to the current suggested Air Quality Guidelines (AQGs) 24-hour interim target 3 (i.e., 37.5 µg/m^3^) (33)). We also plotted a series of model deviances for the association between the average PM2.5 concentration and IVF outcomes and found that the minimum deviance values were greater than 40 µg/m^3^ (**Supplementary Figure 1**). Therefore, we used the 24-hour interim target 2 (50 µg/m^3^) (34) as a threshold for excessive PM2.5 exposure (24).

Multinomial logistic regression analysis was conducted to determine the adjusted odds ratios (AORs) and their corresponding 95% confidence intervals (95% CIs) for the associations between PM2.5 exposure and pregnancy outcomes among women receiving infertility treatments. PM2.5 was included in the models as a continuous variable representing concentrations exceeding 50 μg/m³, and the AORs represented the change in odds of adverse outcomes per 10 μg/m³ increase in PM2.5 above this threshold. To address potential confounding factors, we adjusted for both known and potential variables in our multinomial regression models, as these variables have been identified as potential risk factors for IVF pregnancy outcomes (35, 36). The previous study have suggested that clinical pregnancy and live birth rates declined with each millimeter below 8 mm in fresh IVF-ET and below 7 mm in frozen-ET (37). To validate the estimates, we performed sensitivity analyses by excluding women who underwent blastocyst transfer. R statistical software version 3.6.3, with the ‘nnet’ package used in multinomial logistic regression, was used for conducting the statistical analyses. A two-tailed *p* value of less than 0.05 was considered to indicate statistical significance.

## Results

A total of 2,839 women whose mean age was 32.7 ± 3.9 years were included in the final data analysis. The majority (69.1%) of participants were treated with fresh embryo transfer, while 30.9% received frozen embryo transfer. **Table 1** displays the demographic and clinical characteristics of the participants using descriptive statistics. According to pregnancy outcomes, participants were classified into five groups: full-term birth (34.0% of cases), preterm birth (5.9% of cases), miscarriage (6.4% of cases), biochemical pregnancy loss (6.5% of cases), and nonpregnancy (47.0% of cases).

**Table 1:** Demographic characteristics of couples who underwent IVF with different outcomes.

Variables	All cases (N=2839)	Full-term birth (N=966)	Preterm birth (N=169)	Miscarriage (N=182)	Biochemical pregnancy loss (N=185)	Non-pregnancy (N=1337)	*p*-value
**Female age (years)**	32.7 ± 3.9	31.9 ± 3.3	31.7 ± 3.1	33.4 ± 4.4	32.4 ± 3.9	33.3 ± 4.1	**< 0.001**
**Male age (years)**	34.7 ± 5.2	34.1 ± 4.7	33.4 ± 4.2	35.5 ± 6.0	34.6 ± 4.8	35.3 ± 5.5	**< 0.001**
**Female BMI (kg/m^2^)**							0.445
Underweight	283 (10.2)	111 (11.7)	16 (9.9)	13 (7.3)	13 (7.1)	130 (9.9)	
Normal weight	1594 (57.3)	544 (57.4)	88 (54.3)	102 (57.3)	105 (57.4)	755 (57.7)	
Overweight/Obesity	903 (32.5)	293 (30.9)	58 (35.8)	63 (35.4)	65 (35.5)	424 (32.4)	
**Education level of female**							0.156
High school or below	299 (10.5)	80 (8.3)	18 (10.7)	16 (8.8)	26 (14.1)	159 (11.9)	
Tertiary education	2138 (75.4)	745 (77.2)	127 (75.1)	138 (75.8)	138 (74.6)	990 (74.2)	
Postgraduate education	398 (14.1)	140 (14.5)	24 (14.2)	28 (15.4)	21 (11.4)	185 (13.9)	
**Type of infertility**							0.077
Primary infertility	1821 (64.2)	648 (67.2)	116 (68.6)	111 (61.7)	112 (60.9)	834 (62.4)	
Secondary infertility	1014 (35.8)	317 (32.8)	53 (31.4)	69 (38.3)	72 (39.1)	503 (37.6)	
**Factors of infertility**							0.168
Male	656 (23.2)	230 (23.9)	44 (26.2)	37 (20.6)	46 (25.1)	299 (22.4)	
Female	1627 (57.5)	535 (55.6)	89 (53.0)	119 (66.1)	109 (59.7)	775 (58.1)	
Both	311 (11.0)	103 (10.7)	23 (13.7)	12 (6.7)	19 (10.4)	154 (11.5)	
Unknown	234 (8.3)	95 (9.9)	12 (7.1)	12 (6.7)	9 (4.9)	106 (7.9)	
**Duration of infertility (years)**	3.1 ± 2.2	3.0 ± 2.1	3.0 ± 2.2	3.1 ± 2.6	3.0 ± 2.1	3.2 ± 2.3	0.325
**Gonadotropin doses (IU)**	1800 (900)	1800 (825)	1875 (900)	1800 (816)	1931 (1050)	1875 (975)	0.214
**Duration of stimulation (days)**	10 (4)	10 (3)	10 (3)	10 (4)	10 (4)	10 (3)	**0.002**
**Stimulation protocol**							**< 0.001**
GnRH agonist	1483 (52.2)	553 (57.2)	113 (66.9)	76 (41.8)	97 (52.4)	644 (48.2)	
GnRH antagonist	710 (25.0)	225 (23.3)	29 (17.2)	45 (24.7)	45 (24.3)	366 (27.4)	
Other protocols	646 (22.8)	188 (18.5)	27 (15.9)	61 (33.5)	43 (23.2)	327 (24.5)	
**Number of retrieved oocytes**	10.6 ± 6.4	11.1 ± 6.1	11.2 ± 5.9	10.7 ± 7.6	10.6 ± 6.5	10.1 ± 6.4	**0.003**
**Fertilization method**							0.216
IVF	1939 (68.3)	670 (69.4)	120 (71.0)	131 (72.0)	115 (62.2)	903 (67.5)	
ICSI	900 (31.7)	296 (30.6)	49 (29.0)	51 (28.0)	70 (37.8)	434 (32.5)	
**Rate of fertilization (%)**	73.3 ± 22.2	73.8 ± 21.7	75.4 ± 21.3	74.5 ± 21.7	76.7 ± 18.8	72.1 ± 23.1	**0.021**
**Rate of good embryos (%)**	26.6 ± 22.2	29.3 ± 21.5	31.5 ± 23.4	27.8 ± 21.5	29.5 ± 21.4	23.5 ± 22.4	**< 0.001**
**Endometrial thickness (mm)**	11.0 ± 2.2	11.1 ± 2.1	10.9 ± 1.9	10.7 ± 2.1	11.3 ± 2.4	10.9 ± 2.2	**0.010**
**Type of embryo transfer**							**0.008**
Fresh	1961 (69.1)	691 (71.5)	128 (75.7)	111 (61.0)	120 (64.9)	911 (68.1)	
Frozen	878 (30.9)	275 (28.5)	41 (24.3)	71 (39.0)	65 (35.1)	426 (31.9)	
**Year of embryo transfer**							**< 0.001**
2016	403 (14.2)	130 (13.5)	22 (13.0)	29 (15.9)	25 (13.5)	197 (14.7)	
2017	726 (25.6)	279 (28.9)	57 (33.7)	35 (19.2)	60 (32.4)	295 (22.1)	
2018	811 (28.5)	295 (30.5)	72 (42.6)	46 (25.3)	47 (25.4)	351 (26.3)	
2019	899 (31.7)	262 (27.1)	18 (10.7)	72 (39.6)	53 (28.6)	494 (36.9)	
**Number of transferred embryos**							**< 0.001**
One	1192 (42.0)	335 (34.7)	18 (10.7)	73 (40.1)	72 (38.9)	694 (51.9)	
Two	1647 (58.0)	631 (65.3)	151 (89.3)	109 (59.9)	113 (61.1)	643 (48.1)	
**Stage of transferred embryos**							0.082
Blastocyst	135 (4.8)	38 (3.9)	3 (1.8)	12 (6.6)	8 (4.3)	74 (5.5)	
Cleavage	2704 (95.2)	928 (96.1)	166 (98.2)	170 (93.4)	177 (95.7)	1263 (94.5)	

SD, standard deviation; BMI, body mass index; IQR, interquartile range.

Bold values are statistically significant.

The daily PM2.5 concentrations of the participants during different periods are shown in **Table 2**. There were significant differences in the average daily PM2.5 concentration among women with distinct pregnancy outcomes during Period 4 (from embryo transfer to the serum hCG test), Period 6 (after confirmation of clinical pregnancy), and Period 7 (after confirmation of biochemical pregnancy). The results displayed in **Table 3** demonstrate a significant correlation between each 10 µg/m^3^ increase in PM2.5 above the threshold of 50 µg/m^3^ and increased risk of miscarriage (AOR = 2.22; 95% CI: 1.75-2.81 for Period 6; AOR = 2.23; 95% CI: 1.68-2.96 for Period 7), using full-term birth as the reference group (**Table 3**). In addition, excessive PM2.5 pollution above the 50 µg/m^3^ threshold was associated with a 46% increase in the risk of preterm birth (AOR = 1.46, 95% CI: 1.09-1.94) during period 6 and a 61% increase in the risk of preterm birth (AOR = 1.61, 95% CI: 1.16-2.23) during period 7.

**Table 2:** Description of the PM2.5 concentration in different periods among IVF patients.

	All cases (N=2839)	Full-term birth (N=966)	Preterm birth (N=169)	Miscarriage (N=182)	Biochemical pregnancy loss (N=185)	Non-pregnancy (N=1337)	*p*-value
**Period 1**	37.0 ± 9.4	37.2 ± 9.3	37.5 ± 9.0	37.1 ± 9.6	36.5 ± 9.4	36.8 ± 9.5	0.741
**Period 2**	37.0 ± 12.9	37.0 ± 12.3	37.0 ± 13.2	37.3 ± 13.1	37.6 ± 12.7	36.9 ± 13.2	0.965
**Period 3**	37.0 ± 8.9	37.2 ± 8.7	37.4 ± 8.5	37.1 ± 9.1	36.6 ± 8.8	36.9 ± 9.1	0.846
**Period 4**	36.9 ± 13.0	38.0 ± 13.0	36.4 ± 13.5	35.7 ± 13.9	37.5 ± 13.9	36.2 ± 12.7	**0.011**
**Period 5**	37.6 ± 16.1	38.1 ± 16.2	37.1 ± 15.1	36.1 ± 15.1	37.1 ± 17.2	---	0.366
**Period 6**	36.6 ± 6.6	36.7 ± 4.9	37.6 ± 6.0	34.7 ± 12.4	---	---	**0.015**
**Period 7**	36.6 ± 6.2	36.8 ± 4.7	37.6 ± 5.8	34.8 ± 11.2	---	---	**0.010**

Bold values are statistically significant.

**Table 3:** Associations of daily PM2.5 above the WHO interim target 2 in different periods with IVF outcomes among participants in Shanghai.

	Preterm birth (N=169)	Miscarriage (N=182)	Biochemical pregnancy loss (N=185)	Non-pregnancy (N=1337)
	AOR (95% CI) ^a^*	AOR (95% CI) ^a^*	AOR (95% CI) ^a^*	AOR (95% CI) ^a^*
**Period 1**	0.93 (0.83-1.04)	0.97 (0.87-1.09)	0.97 (0.87-1.09)	0.97 (0.91-1.03)
**Period 2**	1.04 (0.96-1.12)	1.04 (0.97-1.12)	1.02 (0.95-1.10)	1.03 (0.99-1.07)
**Period 3**	1.00 (0.88-1.12)	0.97 (0.85-1.10)	1.01 (0.89-1.13)	1.00 (0.93-1.07)
**Period 4**	0.94 (0.87-1.02)	0.97 (0.90-1.05)	0.99 (0.92-1.06)	0.97 (0.93-1.00)
**Period 5**	0.99 (0.93-1.06)	0.97 (0.90-1.04)	1.03 (0.97-1.09)	---
**Period 6**	**1.46 (1.09-1.94)**	**2.22 (1.75-2.81)**	---	---
**Period 7**	**1.61 (1.16-2.23)**	**2.23 (1.68-2.96)**	---	---

AOR, adjusted odds ratio; 95% CI, 95% confidence interval.

*Full-term birth was used as the reference level, and the AOR is for each 10 µg/m³ increment of ambient PM2.5.

a Adjusted for the age of the male and female, type of infertility, factors of infertility, duration of stimulation, stimulation protocol, number of retrieved oocytes, rate of good embryos, thickness of the endometrium, type of embryo transfer, year of embryo transfer, number of transferred embryos, stage of transferred embryos, and rate of fertilization.

Bold values are statistically significant.

**Figure 3:**
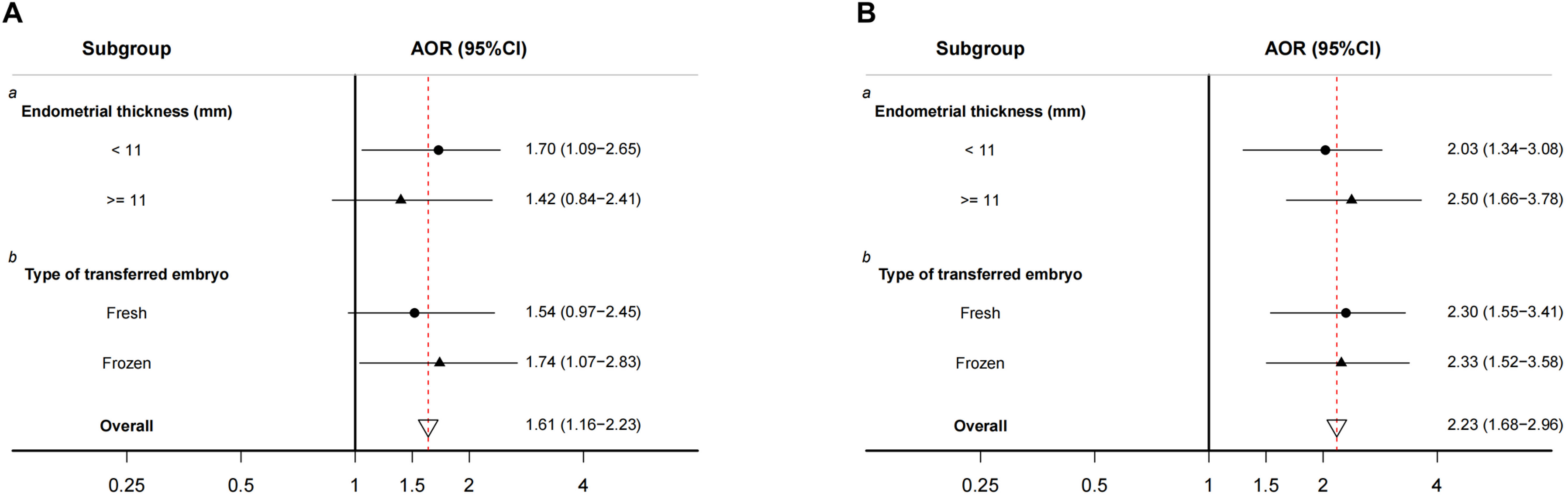
Stratified analyses of associations between daily PM2.5 above the WHO interim target 2 in period 7, **(A)** preterm birth and **(B)** miscarriage. AOR, adjusted odds ratio; 95% CI, 95% confidence interval. The full-term birth group was used as a reference. AOR for each 10 µg/m³ increment of ambient PM2.5. ^a^ Adjusted for the age of male and female, type of infertility, factors of infertility, duration of stimulation, stimulation protocol, number of retrieved oocytes, rate of good embryos, type of embryo transfer, year of embryo transfer, number of transferred embryos, stage of transferred embryos, and rate of fertilization. ^b^ Adjusted for the age of the male and female, type of infertility, factors of infertility, duration of stimulation, stimulation protocol, number of retrieved oocytes, rate of good embryos, thickness of the endometrium, year of embryo transfer, number of transferred embryos, stage of transferred embryos, and rate of fertilization.

We examined whether the estimated associations between PM2.5 and preterm birth and miscarriage differed among subgroups in Period 7. As shown in **Figure 3**, the estimated association with preterm birth varied by endometrial thickness and type of embryo transfer. Women with an endometrial thickness <11 mm or frozen embryo transfer were more susceptible to the adverse effects of a 10 µg/m^3^ increase in PM2.5 above the threshold on preterm birth rates; however, there was no significant variation in the association between excessive PM2.5 exposure and miscarriage among these subgroups for Period 7. The association remained almost unchanged in the sensitivity analyses (**Supplementary Table 1**).

## Discussion

The findings of our study suggest a significant association between maternal exposure to high levels of ambient PM2.5 (above 50 µg/m^3^) after confirmation of biochemical pregnancy or clinical pregnancy and increased risks of miscarriage and preterm birth in women who underwent ART treatment. Moreover, a significant association between PM2.5 exposure and miscarriage was observed in all subgroups, covering diverse endometrial thickness ranges and types of embryo transfer, whereas the detrimental effects on preterm birth were more pronounced among women with endometrial thickness <11 mm or who underwent frozen embryo transfer.

The relationships between exposure to PM2.5 and adverse pregnancy outcomes, such as preterm birth and pregnancy loss, among women undergoing IVF treatment are still not fully understood. The existing research presents varying findings, with some studies suggesting a potential association between PM2.5 exposure and adverse outcomes, while others do not find significant correlations (12, 19, 23, 25). Factors such as the concentration and duration of PM2.5 exposure, as well as geographical location, may influence the outcomes observed. Studies conducted in areas with higher levels of PM2.5 pollution tend to show a stronger association with adverse pregnancy outcomes (13, 23, 25).

Only a few population-based studies have examined the effects of maternal exposure to PM2.5 on miscarriage (38). A retrospective cohort study conducted in a naturally conceived population demonstrated that miscarriage was associated with maternal acute exposure to ambient PM2.5 during the four weeks after conception (38). Furthermore, a significant relationship was observed between PM2.5 and fetal death caused by miscarriage (39). However, the effect of PM2.5 exposure in the ART population on the incidence of miscarriage is unclear. We found that ambient PM2.5 exposure after the confirmation of biochemical or clinical pregnancy was associated with a greater risk of miscarriage in infertile women undergoing IVF cycles. In contrast, two multicenter retrospective cohort studies in the USA and China suggested that PM2.5 was not associated with a decreased risk of live birth or increased risk of pregnancy loss in women who underwent ART (18, 20). The discrepancy in the literature may be due to differences in study design (exposure at patients’ address or IVF unit), air pollution monitoring mode (monitoring station or different validated models), different IVF cycle protocols among the centers, PM2.5 concentrations and organic composition (high or low in different regions), and demographic characteristics of the study population.

The global number of preterm births associated with PM2.5 was estimated to be approximately 2.7 million in 2010 (40). Preterm birth is a major contributor to perinatal and early neonatal mortality and has been linked to adverse long-term health consequences, including cognitive, immunological, neurodevelopmental, and cardiovascular diseases (3). With the development of infertility treatment, the goal is to help infertile couples obtain healthy babies, not just live births. Therefore, it is crucial to investigate and understand the effects of PM2.5 on preterm birth among the population undergoing IVF treatment. A study in the USA reported that a 10% decrease in PM2.5 levels nationwide in 2008 contributed to a reduction of 5,016 preterm births, potentially reducing government expenditures by hundreds of millions of dollars annually, and that when additional health expenditures for preterm birth offspring in later years are taken into account, the savings in health finances could exceed $1 billion (41). Furthermore, a national cohort study in mainland China demonstrated that women who underwent IVF treatment had a greater risk of preterm birth due to PM2.5 exposure during the third trimester than women who conceived naturally (12). Although the local government has implemented stringent policies to reduce PM2.5 levels, exposure to unhealthy levels of PM2.5 is common for Shanghai residents (42). In this study, we found that a high level of PM2.5 exposure above the WHO interim target 2 (50 µg/m^3^) during pregnancy was significantly related to increased odds of preterm birth following the IVF cycle. However, Shi et al. reported a significant link between PM2.5 exposure and a greater rate of preterm birth within 85 days before oocyte retrieval, from the onset of gonadotropin administration until oocyte retrieval, throughout the first trimester of pregnancy, and throughout the entire IVF pregnancy (23). A potential explanation for this might be that the percentage of two embryos transferred in their study was as high as 84.5% (58% in our study), and the preterm birth rate in their study was much greater than that in our study (9.25% *versus* 5.95%). Previous research has shown an association between embryo transfer and an increased risk of preterm birth (43, 44). Consequently, transferring multiple embryos as an independent risk factor for preterm birth may influence the outcomes of PM2.5 exposure.

Stratified analyses provide additional evidence that excessive PM2.5 exposure over the threshold (i.e., 50 µg/m^3^) may act together with other biological factors (e.g., endometrial thickness) and clinical treatment (e.g., the type of embryo transfer) to induce systemic inflammation and affect pregnancy duration (45, 46). A thin endometrium is associated with poor vascularization and reduced blood supply to the placenta, resulting in increased oxidative stress and inflammatory reactions (47). As a result, this condition can potentially compromise the process of placentation and hinder fetal growth, thereby contributing to adverse maternal and perinatal outcomes (48). Our observations indicated that subjects who had an endometrial thickness <11 mm rather than an endometrial thickness ≥ 11 mm had a greater risk of preterm birth when exposed to PM2.5 after biochemical pregnancy. In addition, our findings indicate that the adverse effects of transferring frozen embryos might contribute to an increased susceptibility to preterm birth when exposed to high levels of PM2.5. Retrospective studies suggested that frozen embryo transfer significantly increased the chance of preterm birth and abnormal placentation even after adjusting for BMI, maternal age and other confounders (49, 50). The combination of frozen embryo transfer and exposure to high levels of PM2.5 may have a synergistic effect, further increasing vulnerability to preterm birth. The underlying mechanisms of this interaction are not fully understood but may involve the compromised uterine environment and altered immune responses associated with frozen embryo transfer (51). Therefore, women who bear these risk factors may be a specific target group for future interventions and preventive measures.

Evidence from animal models suggests that mitochondrial dysfunction, oxidative stress, inflammation, and epigenetic alterations could be the underlying mechanisms of the reproductive toxicity of PM2.5 (52–54). High doses of PM2.5 induce the apoptosis of ovarian granulosa cells and oocytes and disrupt embryo development and impair placentation (54). PM2.5 was also associated with more severe lipid peroxidation and inadequate antioxidant capacity in women who suffer a miscarriage than in the normal pregnant population (55). Exposure to PM2.5 might interfere with the transport of oxygen and nutrients and/or accumulation in the placenta to directly cause placental inflammation and senescence, which can lead to preterm birth and miscarriage (56). Further investigation is needed to understand the mechanisms underlying the observed associations between PM2.5 exposure and miscarriage and preterm birth.

There are several strengths in our study. To our knowledge, this is one of the few studies to examine the relationship between PM2.5 exposure and adverse pregnancy outcomes in ART population. Our findings suggest that PM2.5 exposure is associated with a considerable burden of miscarriage and preterm birth among women undergoing infertility treatment. By avoiding outdoor activities in areas with air pollution, pregnant women who undergo IVF treatment may reduce their exposure to PM2.5 particles and mitigate the associated risks to their pregnancy. In addition, previous cohort studies were mainly conducted in women who conceived naturally (2, 7, 8), for whom it is challenging to identify the specific susceptible exposure. Instead, the stage of pregnancy during ART treatment is clearly recorded (e.g., dates of gonadotropin stimulation, embryo transfer, hCG test, and ultrasound examinations). This study used a characterized model with well-defined exposure timelines to study potential susceptible windows, which provides important insights for further study. Moreover, the satellite-based PM2.5 exposure assessment in our study could provide more detailed and additional useful information than the ground-based exposure assessment (57).

There are a few limitations of our study. First, this study analyzed only embryo transfer cycles, excluding cycles that were terminated or without viable embryos, potentially causing selection bias. The explanation for this is that this particular population exhibits the most serious consequences of IVF treatment, making it difficult to determine the impact of PM2.5 pollution on these occurrences. However, this selection bias may cause the adverse effects of PM2.5 to decrease to null values. Second, our data were obtained from medical records as a retrospective study, containing only limited information on individual covariates; thus, residual confounding effects could not be completely ruled out. Third, the absence of details regarding the utilization of fresh air filtration could result in misclassification of the exposure. Hence, it is necessary to conduct multicenter prospective studies to validate the results of this study.

## Conclusions

This study demonstrated that women who received ART and who were exposed to PM 2.5 after pregnancy had a greater risk of preterm birth and miscarriage. Women with an endometrial thickness <11 mm or who underwent frozen embryo transfer were more susceptible to increased risks of preterm birth associated with PM2.5. Additionally, an association between PM2.5 exposure and miscarriage after pregnancy was found in all subgroups, encompassing a wide range of endometrial thickness levels and various types of embryo transfer. Nevertheless, the underlying mechanism of the adverse effects of PM2.5 on pregnancy outcomes among women undergoing ART needs further investigation.

## Data Availability

The original contributions presented in the study are included in the article/**Supplementary Material**. Further inquiries can be directed to the corresponding authors.
