# Supplementary material for: Associations of maternal PM2.5 exposure with preterm birth and miscarriage in women undergoing in vitro fertilization: a retrospective cohort study
Source: Front Endocrinol (Lausanne). 2025 Jan 27;16:1460976. doi: 10.3389/fendo.2025.1460976 (PMC11808209; doi:10.3389/fendo.2025.1460976)
Supplement: Supplementary file 1 [file DataSheet1.docx]

**Supplementary figure 1. Deviances of models estimating the associations between different PM2.5 concentrations (µg/m³) and IVF outcomes**

**
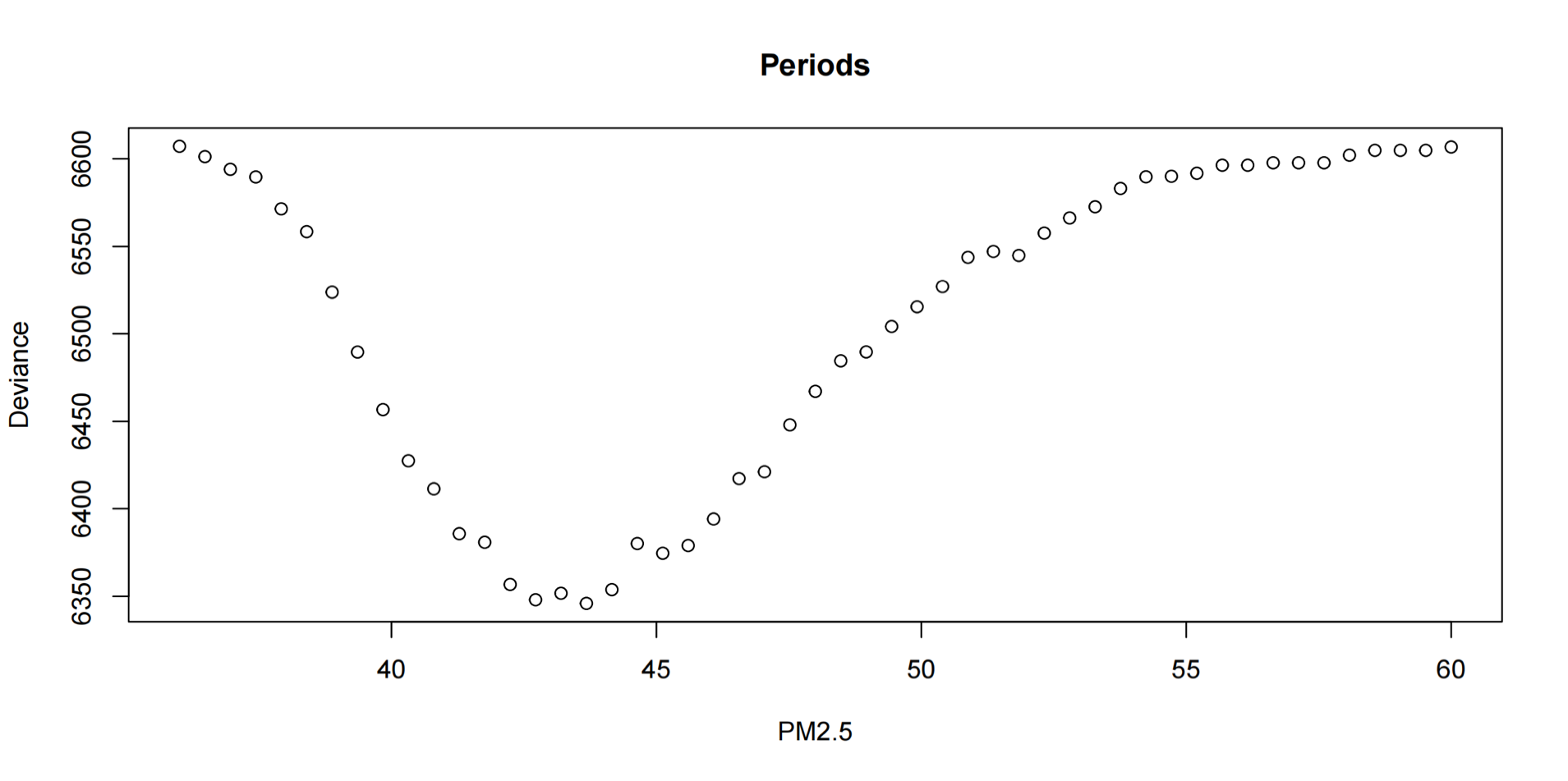
**

The results were adjusted for male and female age, infertility type, duration of stimulation, stimulation protocol, number of retrieved oocytes, rate of good embryos, thickness of the endometrium, type of embryo transfer, year of embryo transfer, number of transferred embryos, stage of transferred embryos, and rate of fertilization.

**Supplementary Table 1. Sensitivity analysis of the association between daily PM2.5 concentrations above the WHO interim target 2 and IVF outcomes among patients who underwent embryo cleavage**

|  | **Preterm birth**  **(N=166)** | **Miscarriage**  **(N=170)** | **Biochemical pregnancy loss**  **(N=177)** | **Non-pregnancy**  **(N=1263)** |
| --- | --- | --- | --- | --- |
|  | AOR (95% CI) ^a^ | AOR (95% CI) ^a^ | AOR (95% CI) ^a^ | AOR (95% CI) ^a^ |
| **Period 1** | 0.94 (0.83-1.05) | 0.98 (0.88-1.11) | 0.98 (0.88-1.10) | 0.98 (0.92-1.04) |
| **Period 2** | 1.04 (0.96-1.12) | 1.03 (0.96-1.11) | 1.01 (0.93-1.08) | 1.03 (0.99-1.07) |
| **Period 3** | 1.01 (0.89-1.14) | 0.98 (0.86-1.12) | 1.02 (0.90-1.15) | 1.01 (0.94-1.08) |
| **Period 4** | 0.95 (0.88-1.02) | 0.97 (0.90-1.05) | 0.99 (0.92-1.06) | 0.97 (0.93-1.01) |
| **Period 5** | 0.99 (0.93-1.06) | 0.97 (0.90-1.04) | 1.03 (0.97-1.09) | --- |
| **Period 6** | **1.46 (1.09-1.94)** | **2.20 (1.73-2.79)** | --- | --- |
| **Period 7** | **1.61 (1.16-2.23)** | **2.22 (1.67-2.95)** | --- | **---** |

AOR, adjusted odds ratio; 95% CI, 95% confidence interval.

The full-term birth group was used as a reference. AOR for each 10 µg/m³ increment of ambient PM2.5.

^a^ Adjusted for male and female age, infertility type, duration of stimulation, stimulation protocol, number of retrieved oocytes, rate of good embryos, thickness of the endometrium, type of embryo transfer, year of embryo transfer, number of transferred embryos, and rate of fertilization.
